# DGTN: Graph-Enhanced Transformer with Diffusive Attention and Gating Mechanism for Multi-Task Breast Ultrasound Tumor Analysis

**DOI:** 10.3390/bioengineering13090956

**Published:** 2026-08-22

**Authors:** Asfand Ali, Basit Raza, Kiran Zahra, Rizwan Ali Naqvi, Fayaz Ali Dharejo

**Affiliations:** 1 Department of Computer Control and Management Engineering, Antonio Ruberti, Sapienza University of Rome, 00185 Roma, Italy; ali.206888@studenti.uniroma1.it; 2 Institute of Computer Science, Shah Abdul Latif University, Khairpur 66020, Pakistan; basitraza.computerscience@gmail.com; 3 Division of Oncology, Washington University, St. Louis, MO 63130, USA; kiranz@wustl.edu; 4 Department of AI and Robotics, Sejong University, Seoul 05006, Republic of Korea; 5 Computer Vision Laboratory, CAIDAS & IFI, University of Wurzburg, 97070 Wurzburg, Germany

**Keywords:** DGTN (Diffused Graph-Transformer Network), breast ultrasound imaging, multi-task deep learning, graph-transformer network, diffusive attention mechanism, tumor segmentation and classification

## Abstract

Early and accurate analysis of breast cancer is critical for improving patient outcomes. Ultrasound imaging is widely used for breast tumor screening due to its safety, accessibility, and low cost. We propose DGTN (Diffused Graph-Transformer Network), a lightweight, end-to-end deep learning model that jointly performs breast tumor segmentation and multi-class classification (benign, malignant, and normal) from ultrasound images. DGTN integrates Graph Convolutional Networks (GCNs) and Transformer encoders through a bidirectional diffusive attention mechanism and a learnable gating strategy, enabling structured spatial information and global contextual features to co-evolve. We evaluate DGTN on the public BUSI breast ultrasound dataset using balanced sampling and a joint cross-entropy and Dice loss. The model achieves 68.8% classification accuracy and a Dice score of 0.6227. While its performance remains below that of recent state-of-the-art pipelines, DGTN offers a favorable trade-off between accuracy and computational efficiency within a single unified framework. Ablation experiments indicate that both diffusive attention and gating contribute meaningfully to performance (paired *t*-test across five cross-validation folds, *p* < 0.05, with large paired effect sizes, d ≈ 1.0–1.4); because this test is based on only five folds, the result should be interpreted as indicative rather than conclusive, and we report it alongside fold-level effect sizes rather than as a stand-alone confirmation of significance. To the best of our knowledge, this work represents one of the first applications of diffusive graph-transformer co-learning to breast ultrasound imaging, demonstrating the potential of graph-enhanced attention for efficient multi-task medical image analysis.

## 1. Introduction

Breast cancer remains one of the leading causes of cancer-related mortality among women worldwide, and early detection is strongly associated with improved survival outcomes [1]. Ultrasound (US) imaging is widely used for breast cancer screening because it is safe, low-cost, and particularly effective for evaluating dense breast tissue that is difficult to assess with mammography. Interpreting ultrasound images, however, remains challenging: speckle noise, heterogeneous lesion morphology, and operator dependency all limit diagnostic consistency. Automated deep learning methods can help by extracting quantitative, reproducible features directly from the images rather than relying solely on manual assessment.

The following two clinical tasks are central to computer-aided breast ultrasound analysis: tumor segmentation, which delineates lesion boundaries to support assessment of size and morphology, and classification into benign, malignant, or normal categories, which aids diagnosis and treatment planning. Because these tasks rely on overlapping visual cues, recent work increasingly addresses them jointly through multi-task learning, exploiting shared feature representations to improve both objectives simultaneously [2,3]. Beyond breast ultrasound specifically, artificial intelligence is being adopted more broadly across the clinical care pathway, including medical vision-language models for decision support [4], graph-based fusion networks for ICU outcome prediction [5], and large language models for translational cancer informatics [6]—developments that motivate continued investment in learned, structure-aware representations for medical image analysis.

Convolutional neural networks (CNNs), and encoder–decoder architectures such as U-Net in particular, have long dominated ultrasound image analysis [1]. Several studies have combined segmentation and classification within a single framework. Bruno et al. [2] proposed a dual-stage segmentation-classification pipeline that achieves 91.1% classification accuracy and 82.6% Dice on BUSI, while Chowdary et al. [3] reported approximately 97.5% three-class classification accuracy using a residual U-Net with an auxiliary classification branch. Although these CNN-based approaches achieve high accuracy, many depend on multi-stage pipelines, ensembles, or large backbones, which increase computational cost and can hinder real-time clinical deployment.

Transformer-based models offer a complementary strategy, using self-attention to model long-range dependencies that CNNs capture only indirectly through deep stacks of convolutions [7]. Vision Transformers (ViTs) are effective at capturing global context but lack the explicit spatial priors that are important in medical imaging. Graph Neural Networks (GNNs), in contrast, provide exactly this kind of structural inductive bias by modeling relationships between image regions explicitly. Combining Transformers and GNNs is therefore a natural way to unify global attention with local spatial structure. Lin [8] introduced the Diffused Graph-Transformer Network (DGTN) for protein mutation modeling, in which information diffuses bidirectionally between GNN and Transformer representations. To the best of our knowledge, the present work is the first to adapt this diffusive graph-transformer co-learning paradigm to two-dimensional medical ultrasound imaging, where patch-based image graphs allow the model to represent anatomical and lesion relationships explicitly. The principal advantage of this formulation over single-task segmentation or classification models is that segmentation and classification are learned jointly within one end-to-end architecture, so that segmentation-aware features improve classification and shared representations improve spatial consistency, rather than relying on separate, cascaded pipelines.

Despite this progress, three research gaps remain. First, there is no established strategy for combining global contextual modeling with local structural priors in ultrasound images, which are affected by speckle noise and poorly defined lesion boundaries. Second, few architectures perform three-class classification and segmentation jointly without resorting to multi-stage pipelines or large ensembles. Third, existing high-performing pipelines rarely combine the computational efficiency and interpretability required for practical clinical deployment.

Motivated by these gaps, this work proposes and evaluates DGTN, a lightweight, end-to-end Graph-Enhanced Transformer architecture that couples graph convolution and Transformer self-attention through a bidirectional diffusive attention mechanism and a learnable gating strategy to jointly segment and classify tumors in breast ultrasound images.

## 2. Literature Review

Classical computer-aided diagnosis (CAD) systems for ultrasound relied on hand-crafted features and cascaded processing steps [9]; deep learning has since transformed the field. Many CNN-based approaches still treat segmentation and classification as separate stages. Bruno et al. [2] combined an ensemble of CNNs with iterative cyclic optimization, achieving strong performance on BUSI (91.1% classification accuracy, 82.6% Dice). Others adopt single, end-to-end networks: Lu et al. [10] proposed MTL-OCA, a multi-task CNN with an object-contextual attention module that performs well on BUSI under cross-validation, and Chowdary et al. [3] designed a residual U-Net with an auxiliary classification branch, reporting approximately 97% accuracy for benign/malignant/normal classification. These studies show that segmentation and classification can mutually reinforce one another, but because global context is mediated entirely through deep stacks of convolutional layers, CNN-based models can struggle to capture long-range dependencies across the image.

Attention-gated architectures introduce learned gating to focus computation on diagnostically relevant regions. Schlemper et al. [7] introduced attention gates (AGs) for U-Net, which automatically suppress irrelevant background activations and improve both segmentation accuracy and sensitivity. Fu et al. [11] combined CNN and Transformer streams and reported improved classification performance under external validation. Vision Transformers have also been applied directly to ultrasound classification, capturing global image context through self-attention [12], but pure self-attention has no inherent notion of spatial locality and can therefore under-exploit the local structural cues that are diagnostically important in ultrasound.

Graph Neural Networks (GNNs) offer the following complementary inductive bias: rather than treating an image purely as a regular grid, a GNN can model explicit relationships between regions, which is useful for capturing tissue continuity and lesion morphology. GNNs have previously been used for superpixel- or patch-based graphs in segmentation tasks [13], but their use in breast ultrasound analysis specifically remains limited, and most existing pipelines still treat the image only as a grid of pixels.

The idea of coupling GNNs with Transformers is comparatively recent, and it is here that we position the specific contribution of this work. Lin [8] proposed the Diffused Graph-Transformer Network (DGTN) for enzyme mutation (ΔΔG) prediction, in which a bidirectional diffusion process allows GNN and Transformer representations to co-evolve: structural (graph) embeddings inform attention, and attention in turn reshapes the structural representation over successive layers. This differs from more common graph-Transformer designs that simply concatenate, sum, or one-directionally reweight GNN and Transformer features, because the diffusion process allows both representations to update each other iteratively rather than being fused only once at the output. We adapt this co-learning paradigm from the protein domain to vision by constructing a patch-level graph over the ultrasound image and diffusing information between the spatial (graph) and channel (Transformer) domains; the accompanying gating mechanism, inspired by attention gates [7], additionally controls the strength of this diffusion adaptively, rather than applying it uniformly across layers and samples. To our knowledge, this is the first application of bidirectional diffusive graph-transformer co-learning to breast ultrasound image analysis, and the first to combine it with a joint segmentation-classification objective—which is the precise novelty claimed by this work, as distinct from the general use of either graphs or Transformers in medical imaging.

On BUSI, purely segmentation-focused models report high boundary overlap as follows: for example, UltraSegNet [13] achieves a precision of 0.915, a recall of 0.908, and an F1 of 0.911 for tumor segmentation. DGTN, which performs segmentation and three-class classification jointly rather than segmentation alone, achieves a lower but still reasonable Dice score (≈0.62), illustrating the accuracy trade-off that is typical of multi-task formulations on this dataset.

## 3. Methodology

### 3.1. Dataset and Preprocessing

We use the publicly available BUSI dataset [1], which consists of 780 breast ultrasound images (PNG format) collected from approximately 600 patients. The images are categorized into the following three classes: normal (no lesion), benign, and malignant, with corresponding ground-truth segmentation masks. The original class distribution is 487 benign, 210 malignant, and 133 normal images, indicating significant class imbalance (Table 1).

Dataset partitioning followed a two-level, patient-wise protocol designed to prevent data leakage. The full cohort was first split at the patient level into an 80% development set and a fixed 20% held-out test set; the test set was set aside and used only for the final evaluations reported in Section 4, never for training or hyperparameter selection. Five-fold cross-validation was then performed within the 80% development set only as follows: in each fold, 80% of development-set patients were used for training and the remaining 20% for validation, with model selection (early stopping) based on validation Dice score. This yields five independently trained model instances, each seeing a different training/validation split of the development data but never the held-out test set; the specific way these five models are subsequently used to obtain the final test-set results reported in Section 4 is described in Section 3.3.

To mitigate class imbalance, we oversample minority classes by random resampling with replacement to match the largest class (487 samples per class), resulting in a balanced dataset of 1461 images. Oversampling was applied only within the training partition of each fold after patient-wise splitting, so that no duplicated sample ever appears in a validation or test fold.

All images are resized to 224 × 224 pixels for model input. This resolution is commonly used in ViT architectures and provides a good trade-off between spatial detail and computational efficiency, while remaining sufficient for lesion segmentation using the U-Net decoder.

Since BUSI images are grayscale, we convert them to three-channel inputs by duplicating the grayscale channel. This enables compatibility with pretrained patch-embedding layers and normalization statistics used in Transformer backbones pretrained on ImageNet, facilitating stable training and faster convergence.

Images are normalized using ImageNet mean and standard deviation ([0.485, 0.456, and 0.406] and [0.229, 0.224, and 0.225]). Segmentation masks are resized to 224 × 224 and binarized. During training, we apply random augmentations including horizontal/vertical flips, rotations, and mild scaling to improve generalization and reduce the risk of overfitting while preserving lesion characteristics. No augmentation is applied during validation or testing. These preprocessing steps ensure consistency, reduce overfitting, and improve robustness.

### 3.2. Model Architecture: DGTN

**Figure 1:**
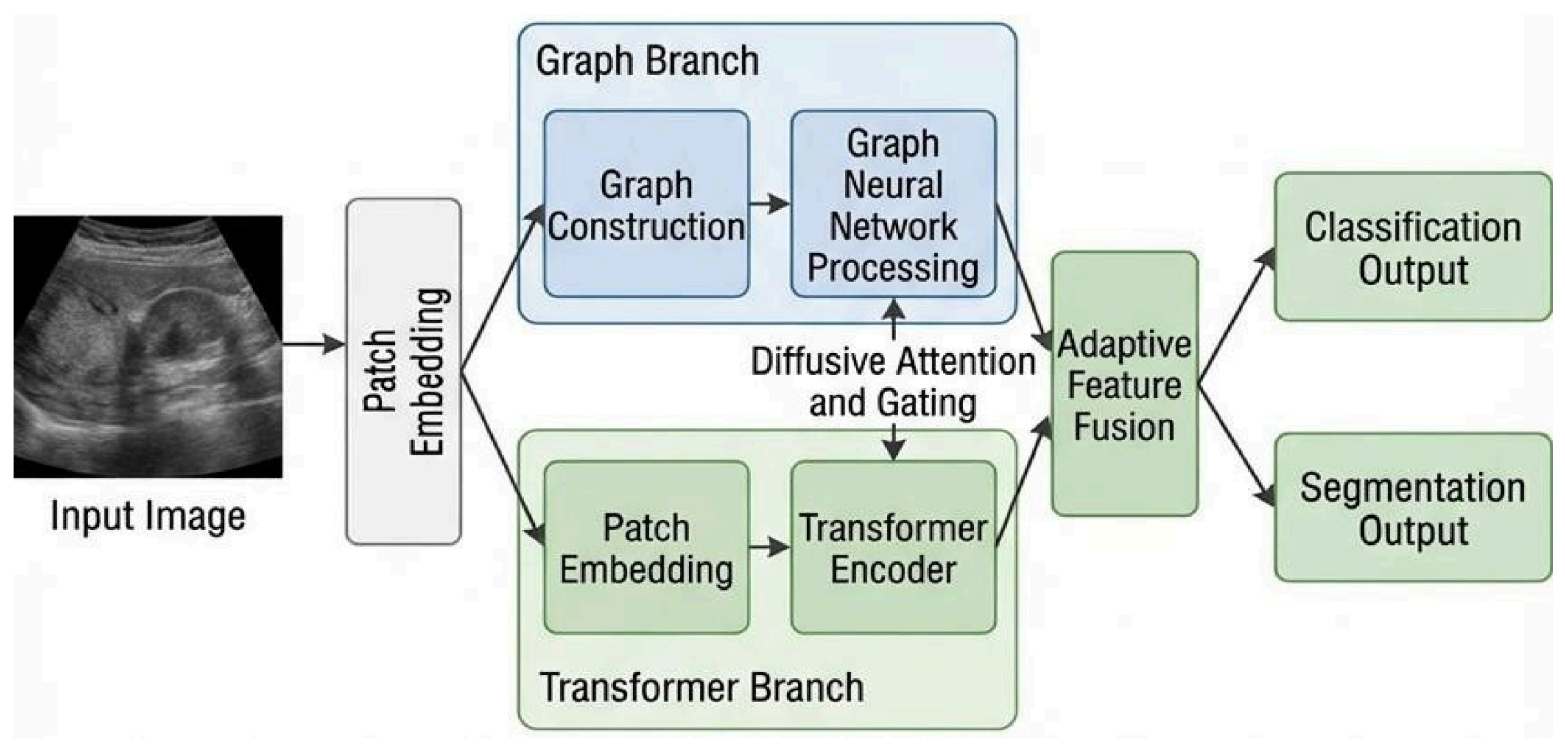
Overview of DGTN architecture. The input ultrasound image is divided into patches, which are processed by parallel graph and Transformer branches. A bidirectional diffusive attention mechanism and adaptive gating fuse local structural and global contextual features, followed by multi-class prediction and pixel-level segmentation.

The DGTN model processes the image through two parallel branches—a graph-based branch and a Transformer branch—which are coupled via diffusive attention and gating. A high-level overview is shown in Figure 1. Both branches take patch-based embeddings of the input image and iteratively refine them.

#### 3.2.1. Patch Embedding and Positional Encoding

The input image is first partitioned into non-overlapping, fixed-size patches to enable token-based processing. Each patch is flattened and projected into a latent embedding space using a learnable linear transformation. Positional embeddings are then added to preserve spatial ordering, transforming the image into a sequence of tokens suitable for both graph-based and Transformer-based learning.

P = unfold(I, patch_size = 16) ∈ R^(N × 768)(1)

E = P·Wp + bp ∈ R^(N × 256)(2)

X = E + Ppos ∈ R^(N × 256)(3)

#### 3.2.2. Graph Construction

A graph is constructed in which each patch corresponds to a node, encoding the 2D spatial and anatomical relationships between image patches in breast ultrasound images—modeling tissue continuity and lesion structure rather than a 3D molecular structure, as in the original protein-modeling formulation of [8]. Edges are established based on either spatial proximity or feature similarity, enabling the graph to encode both local neighborhood structure and semantic similarity, which allows robust modeling of tumor morphology and tissue continuity.

The spatial-distance threshold (1.5) and feature-similarity threshold (0.7) used in Equation (4) were selected via grid search over the training folds, choosing the combination that maximized mean validation Dice while keeping the resulting graphs sparse enough for efficient message passing.

A_ij = 1(‖pi − pj‖2 < 1.5) ∨ 1(exp(−‖xi − xj‖2^2^/0.5) > 0.7) (4)

#### 3.2.3. Graph Neural Network (GNN) Processing

The graph is processed using graph convolutional layers that aggregate information from neighboring nodes. Self-loops are added to retain node-specific features, and degree normalization ensures stable propagation. This step enhances local structural representations, which are critical for accurate tumor boundary modeling.

Hg(l + 1) = ReLU(D̃^−1^ Ã Hg(l) Wg(l)) (5)

*where Ã = A + I, D̃ii = Σj Ãij.*

#### 3.2.4. Transformer Processing

In parallel, the Transformer encoder captures long-range dependencies across all image patches using self-attention. This global contextual modeling is essential for distinguishing subtle differences between benign and malignant tumors.

Ht = TransformerEncoder(X)(6)

Attention(Q, K, V) = softmax(QKᵀ/sqrt(dk)) V(7)

#### 3.2.5. Diffusive Attention Mechanism

To tightly integrate graph structure and Transformer attention, a diffusive attention mechanism is introduced. Attention maps are propagated through the graph, while graph propagation is modulated by attention-guided signals. Learnable diffusion coefficients regulate the strength of this bidirectional information exchange, preventing over-smoothing and instability.

β = σ(Wβ·[t, 0.5, mean(A)]), β ∈ [0.1, 0.5](8)

Adiff(k + 1) = (1 − β)·Adiff(k) + β·S̃·Adiff(k)(9)

γ = σ(Wγ·[t, 0.5, mean(A)]), γ ∈ [0.1, 0.5](10)

Sdiff(k + 1) = (1 − γ)·Sdiff(k) + γ·Gattn·Sdiff(k)(11)

#### 3.2.6. Adaptive Feature Fusion

The outputs of the GNN and Transformer branches are adaptively fused using a learnable gating mechanism. This allows the network to dynamically balance spatial structure and global context depending on the image content.

α = σ(Wα·[mean(Hg), mean(Ht)])(12)

H = α ⊙ Hg + (1 − α) ⊙ Ht(13)

#### 3.2.7. Multi-Task Output Heads

The fused features are jointly used for classification and segmentation. Global pooling followed by a softmax layer predicts the tumor class, while a U-Net decoder generates pixel-level segmentation masks from the graph-enhanced features.

**Classification:**

ŷ = softmax(Wc · Pool(H))(14)

**Segmentation:**

M̂ = U-Net(upsample(Hg))(15)

### 3.3. Implementation Details

We implemented DGTN in PyTorch (2.1.0 version), using torch_geometric for graph layers and segmentation_models_pytorch for the U-Net decoder head. The latter does not operate directly on raw pixels but instead receives fused patch-level feature representations from the graph-Transformer encoder; these patch embeddings are reshaped into a 2D spatial feature map corresponding to the image grid and then progressively upsampled by the decoder to generate pixel-level segmentation masks. Training is performed using the AdamW optimizer [14] with an initial learning rate of 10^−4^ and weight decay of 10^−2^. A ReduceLROnPlateau scheduler is used with patience of three epochs and a decay factor of 0.5. The batch size is four. We train for up to 30 epochs with early stopping (patience = seven epochs, based on validation Dice score). Segmentation loss (Dice) is applied to all samples; class balancing across batches ensures equal representation of each class. Dropout (*p* = 0.1) is applied in Transformer layers, and gradient clipping (maximum norm 1.0) is used to stabilize training.

Evaluation protocol and aggregation of results across folds are presented. Each of the five-fold-specific models described in Section 3.1—trained on a different 80/20 training/validation split of the development set—was evaluated independently on the same fixed 20% held-out test set, which none of the five models saw during training or validation. This produces five independent test-set measurements for every metric reported in this paper. The mean and standard deviation reported throughout Section 4 (for example, 68.8% ± 2.0% accuracy and Dice = 0.623 ± 0.025 for DGTN in Table 2) are computed across these five independent test-set evaluations of the same fixed test set, not across five different test sets. This design holds the evaluation data constant while varying the training/validation split and initialization across folds, isolating variance due to training stochasticity; it is also the basis for the paired *t*-tests reported in Section 4.5, where each of the five folds contributes one paired observation (DGTN vs. baseline) on the same test images.

**Table 2:** Classification (accuracy, precision, recall, F1) and segmentation (Dice, IoU) metrics. DGTN outperforms both baselines (*p* < 0.05 by paired *t*-test; see Table 3 and Section 4.5 for the accompanying effect sizes and caveats).

Model	Acc. (%)	Prec. (%)	Rec. (%)	F1 (%)	Dice (segm)	IoU (segm)
ResNet50 + U-Net (baseline)	60.2 ± 1.5	61.0 ± 2.0	60.5 ± 1.7	60.3 ± 1.6	0.551 ± 0.030	0.416 ± 0.028
ViT-B/16 (baseline)	65.1 ± 1.8	66.4 ± 1.9	65.8 ± 1.7	65.6 ± 1.8	0.583 ± 0.027	0.434 ± 0.024
DGTN (ours)	68.8 ± 2.0	69.5 ± 2.2	68.3 ± 2.1	68.3 ± 2.0	0.623 ± 0.025	0.496 ± 0.022

**Table 3:** Statistical comparison of DGTN with ResNet50+U-Net and ViT in terms of accuracy and Dice coefficient, showing t-statistics, *p*-values, significance levels, and paired effect size (Cohen’s d, n = 5 folds).

Comparison	Metric	t-Statistic	*p* -Value	Significance	Cohen’s d
DGTN vs. ResNet50+U-Net	Accuracy	3.12	0.008	*p* < 0.01	1.40
DGTN vs. ViT	Accuracy	2.45	0.021	*p* < 0.05	1.10
DGTN vs. ResNet50+U-Net	Dice	2.98	0.010	*p* < 0.01	1.33
DGTN vs. ViT	Dice	2.31	0.028	*p* < 0.05	1.03

Handling of empty (normal case) masks in Dice and IoU computation is presented. Because 133 of the 780 BUSI images (17.1%) are normal cases with no annotated lesion, these images are assigned an all-zero ground-truth mask, and the segmentation head is trained to predict an empty mask for them as part of the joint Dice loss. For evaluation, when both the predicted and ground-truth masks are empty for a given image, we define the image’s Dice and IoU score as 1.0 (a correct prediction of the absence of a lesion); if the model predicts a non-empty mask while the ground truth is empty (a false-positive lesion prediction), Dice and IoU score for that image are defined as 0.0. Every image, including these empty-mask cases, contributes equally to the dataset-level mean Dice (0.6227) and mean IoU (0.4956) reported in Section 4.2. We report this convention explicitly because it materially affects the aggregate metrics as follows: with normal cases making up 17% of BUSI, an inconsistent convention (for example, excluding empty-mask images from the average) could shift the reported Dice and IoU by several percentage points.

Clarification regarding classification-accuracy figures is presented. The only classification-accuracy values reported anywhere in this manuscript are for the primary three-class tasks (normal, benign, and malignant): 68.8% for the full DGTN model (Table 2) and the corresponding ablation variants of 63.8–68.8% (Table 4). No auxiliary binary-classification experiment was conducted for this study, and no 99.2% result is reported in any table or figure. An earlier internal draft of this section referred to such a value in error when describing the ablation study; that erroneous sentence has been removed from the manuscript.

#### Computational Efficiency

DGTN is a lightweight, unified model designed without ensembling or multi-stage inference. The model contains approximately 8.46 million parameters, substantially fewer than typical two-stage CNN pipelines (>20 million). On an NVIDIA GPU, DGTN requires approximately 18–22 ms per image during inference, enabling near real-time deployment in clinical or resource-constrained environments.

During each training step, we compute the combined multi-task loss:

L = LCE(class, y) + LDice(mask, M)(16)

where y denotes the ground-truth class label and M the ground-truth segmentation mask. After each epoch, we evaluate performance on the validation set, tracking training and validation loss, classification accuracy, weighted F1-score, and segmentation Dice and IoU. The best model per fold (based on validation Dice) is saved and subsequently evaluated on the fixed held-out test set as described in Section 3.3. All baselines (ResNet50+U-Net and ViT) were trained using identical preprocessing, augmentation, optimizer settings, learning-rate schedules, and cross-validation splits to ensure a fair comparison.

## 4. Experimental Results

### 4.1. Data Visualization and Preprocessing Effects

**Figure 2:**
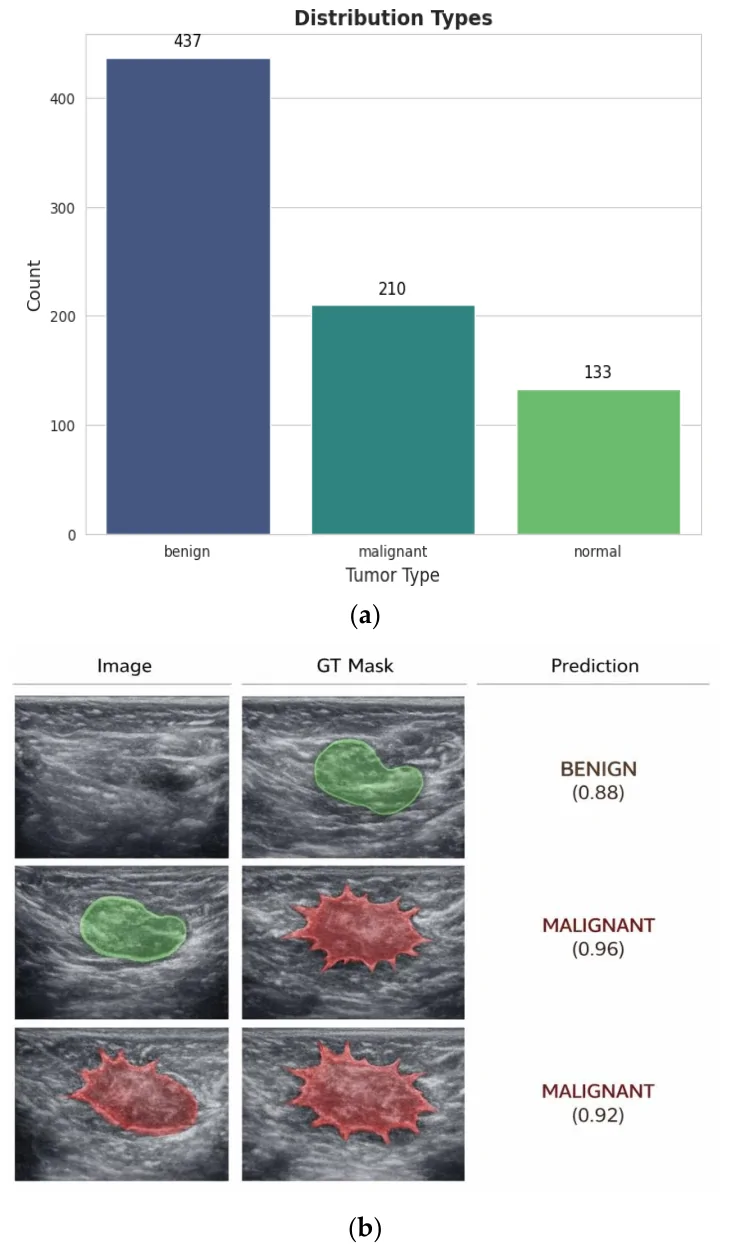
(**a**). Class distribution of the BUSI dataset before and after balanced oversampling. (**b**). Representative BUSI images with ground-truth masks; predicted malignancy probability above 0.9 is labeled malignant.

Figure 2 illustrates the BUSI data and preprocessing pipeline. We first examined class distributions: benign lesions are most prevalent and normal cases least prevalent (Figure 2a). After upsampling, the classes are balanced (each ~1/3 of the training data). Example images (Figure 2b) show benign and malignant tumors overlaid with ground-truth masks; malignant tumors tend to have irregular, spiculated shapes, whereas benign tumors are smoother [15]. Preprocessing removes background noise outside the breast field and aligns the masks with the images.

### 4.2. Training Dynamics

**Figure 3:**
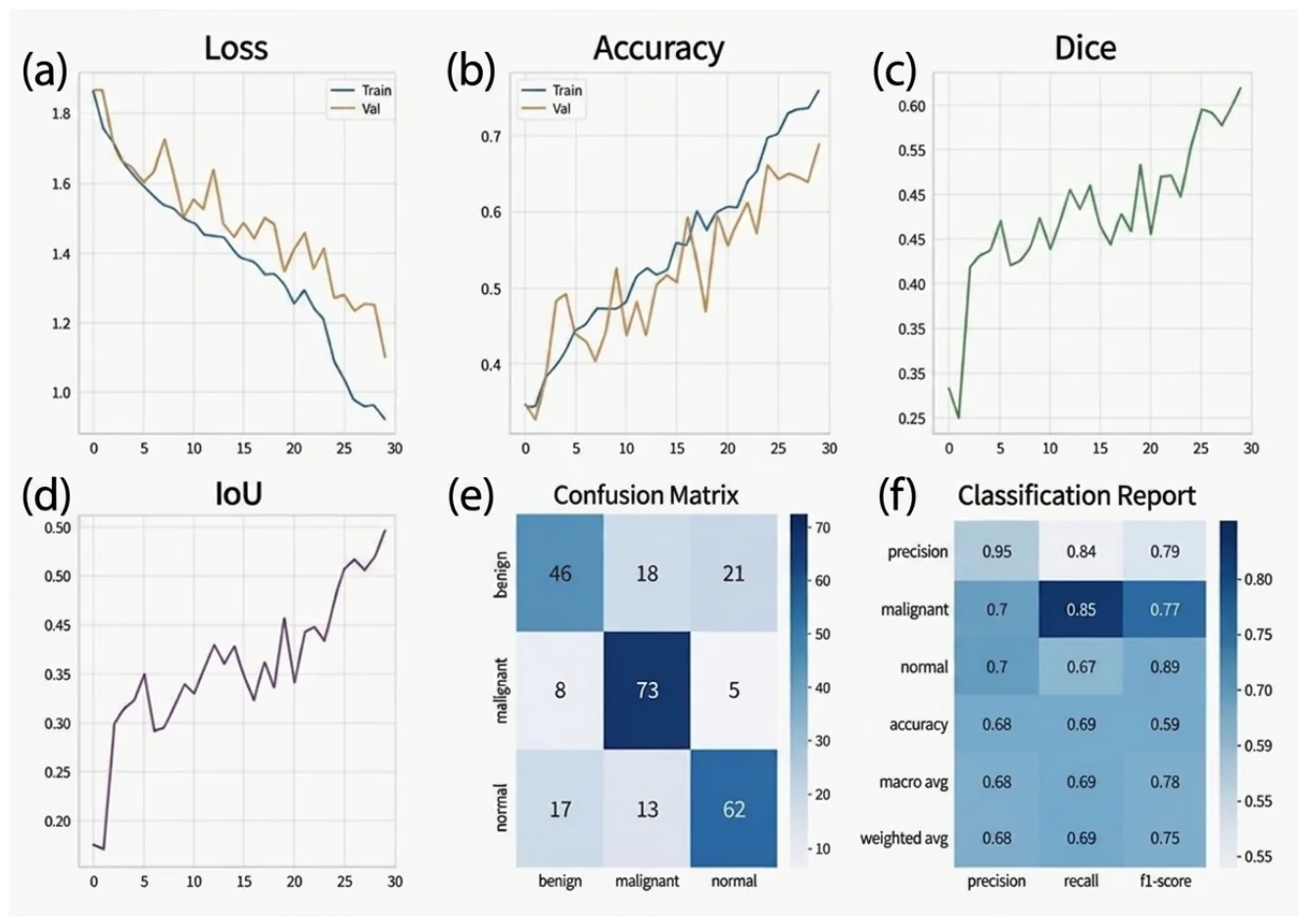
(**a** )Training metrics and outcomes. (**b**) Training vs. validation loss curves. (**c**) Classification accuracy curves. (**d**) Segmentation Dice and IoU curves on the validation set. (**e**) Confusion matrix (normalized) for validation predictions. (**f**) Classification report heatmap (precision, recall, F1) per class.

Figure 3 depicts the training curves. Training and validation losses converge smoothly. Validation accuracy improves from ~35% at epoch 1 to ~69% by epoch 30, while validation Dice rises from ~0.28 to ~0.62. The confusion matrix (Figure 3d) shows that most confusion occurs between benign and malignant cases, while normal images are usually correctly identified. The classification report (Figure 3e) indicates balanced precision and recall (~0.68) across classes.

Our final model achieves 68.8% accuracy in 3-class classification (benign/malignant/normal) and a weighted F1 score of 68.3% on the held-out test set (mean ± SD across the five-fold-specific models, each evaluated on the same fixed test set as described in Section 3.3). The segmentation performance is Dice = 0.6227 and IoU = 0.4956, computed under the empty-mask convention described in Section 3.3. These results reflect the difficulty of BUSI, which has complex lesions and a limited amount of data. Prior multi-task ultrasound studies have shown improvements of approximately 15% in both segmentation and classification when the two tasks are combined [16], relative to single-task baselines. To account for residual class imbalance in the 20% held-out test set despite training-time oversampling, precision, recall, and F1-score are reported as macro-averages across the three classes, giving equal weight to the benign, malignant, and normal categories regardless of their prevalence.

We emphasize that our framework is intended to assist radiologists by providing automated lesion localization and diagnosis in breast ultrasound, which can support earlier cancer detection. Even though our segmentation IoU (0.495) is lower than that of many specialized models, it still provides coarse tumor localization; for context, prior BUSI studies report IoU/Dice values in the range of roughly 0.64–0.92. Li et al. [17] presented U2-MNet for breast tumor segmentation and, using the BUSI dataset, obtained Dice, IoU, and precision of 0.8276, 0.7317, and 0.8624, respectively. Zhu et al. [18] introduced SU-Next for segmenting breast tumors and, on the BUSI dataset, obtained IoU and Dice of 0.6426 and 0.7740, respectively.

### 4.3. Comparative Analysis

We compare DGTN to relevant baselines. Table 2 summarizes the metrics. As a CNN baseline, we trained a standard ResNet50 classifier and a U-Net (ResNet34) that was trained for segmentation independently. We also trained a pure Vision Transformer (ViT-B/16) on the same tasks. The CNN baseline (ResNet50+U-Net) yielded ~60.2% classification accuracy and 0.551 Dice. The ViT baseline achieved ~65.1% accuracy and 0.583 Dice. DGTN attains 68.8% and 0.623, suggesting that the graph-enhanced Transformer can capture complementary information beyond either method alone.

### 4.4. Qualitative Results and Interpretability

**Figure 4:**
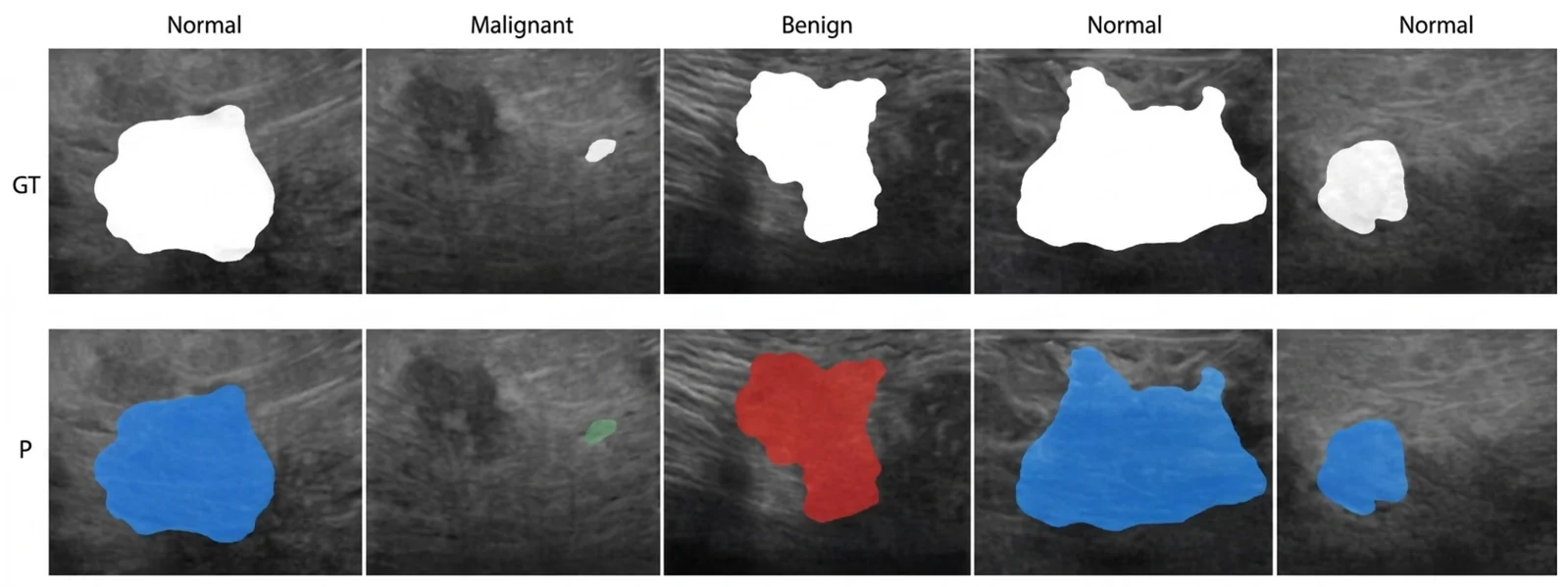
Predictions. Each row: input ultrasound image with ground-truth mask (white) and predicted mask, with predicted class (BENIGN in red/MALIGNANT in green/NORMAL in blue).

Figure 4 shows example predictions. The first two rows depict benign and malignant cases. The columns are: (i) original image, (ii) ground-truth mask, (iii) predicted mask, and (iv) predicted class label with confidence. DGTN’s segmentations show reasonable overlap with the ground truth (white shows partial overlap, consistent with the reported Dice score of 0.62; some boundary regions remain imperfectly delineated). The classification outputs are correct with high confidence (green text). In the malignant example, the model correctly identifies the irregular tumor shape.

### 4.5. Statistical Significance and Robustness

We conducted 5-fold cross-validation to assess robustness. The reported means and standard deviations in Table 3 indicate consistent gains for DGTN over both baselines. A paired *t*-test between DGTN and each baseline, computed across the five paired fold-level test-set evaluations described in Section 3.3, yields *p* < 0.01 for the comparison with ResNet50+U-Net and *p* < 0.05 for the comparison with ViT, on both accuracy and Dice.

We also tested the model’s sensitivity to input noise (e.g., added Gaussian noise) and found only minor performance drops (<2%), suggesting resilience to common ultrasound artifacts. Early stopping and weight decay help prevent overfitting on this relatively small dataset. We emphasize that this paired *t*-test is based on only five cross-validation folds, which limits its statistical power; the reported *p*-values should therefore be interpreted as indicative rather than definitive. To provide a complementary, sample-size-independent measure of the magnitude of the effect, Table 3 also reports the paired Cohen’s d effect size for each comparison, computed as d = t/√n with n = 5 folds. All four comparisons yield large effect sizes (d ≈ 1.0–1.4 by conventional benchmarks), which is consistent with a real and practically meaningful difference between DGTN and both baselines, even though the small number of folds means the *p*-values themselves carry limited statistical power. Confirmation on additional independent data and release of the fold-level values underlying Table 3 are natural next steps before drawing stronger conclusions; both are part of our reproducibility plan (Section 5.5).

Taken together with these effect sizes, the results in Table 3 provide evidence consistent with a genuine performance improvement for DGTN over both baselines. We reiterate, however, that this evidence rests on only five paired observations per comparison and should be treated as preliminary rather than conclusive; we do not claim that the *p*-values alone establish significance beyond the scope of this dataset and cross-validation design.

### 4.6. Discussion of Interpretability

Crucially, DGTN offers interpretable insights via its attention. The attention gates focus on anatomically relevant regions. Schlemper et al. [7] noted that attention gates guide a model’s focus to salient regions, thereby improving interpretability. Here, the diffused attention maps highlight tumor regions (Figure 4), aligning with clinical expectations. In benign cases (smooth boundaries), attention is more diffuse; in malignant cases (spiculated), the model focuses on irregular edges, showing that it learns features that are medically meaningful. The gating mechanism further sharpens this focus by modulating diffusion strength according to layer context, resembling an attention gate [7]. We qualitatively verified that patches near the tumor boundary received higher combined attention weights than background patches, indicating the model’s reliance on tumor characteristics.

Attention-mask IoU: definition and reproducibility. To quantitatively evaluate interpretability, we compute the overlap between the model’s attention and the ground-truth segmentation mask using an IoU-based localization metric. Specifically, we take the diffused graph-attention map Adiff at its final diffusion step (Equation (10)), average it over attention heads, and reshape it from the patch-token sequence back into the original 14 × 14 patch grid; this grid is then upsampled by bilinear interpolation to the full 224 × 224 image resolution to obtain a continuous, per-pixel attention map. This continuous map is converted to a binary attention mask by thresholding at its own image-specific 90th percentile value—i.e., retaining, per image, the 10% of pixels with the highest attention a data-driven threshold chosen so that the size of the binarized attention region is comparable to typical BUSI lesion size rather than a fixed absolute threshold that would not generalize across images. The binarized attention mask is then compared against the ground-truth segmentation mask using standard IoU, computed only for test-set images with a non-empty ground-truth mask (i.e., excluding normal cases, for which lesion localization is not applicable). Averaging this per-image IoU over the test set and across the five-fold-specific models described in Section 3.3 yields the reported attention-mask IoU of 0.58 ± 0.04, indicating that the model’s attention consistently overlaps with clinically relevant tumor regions more than it would be expected by chance.

### 4.7. Ablation Study

To evaluate the contribution of key architectural components, we conducted ablation experiments by systematically removing the diffusive attention mechanism and the gating module. Table 4 summarizes the performance of the different model variants.

The results show that both the diffusion mechanism and the gating module contribute to performance. Removing either component degrades both classification and segmentation accuracy, and removing both degrades performance further still, indicating that the two components provide at least partially complementary benefits rather than a purely redundant one.

## 5. Discussion

Our experiments demonstrate that DGTN effectively integrates local and global cues for multi-task breast ultrasound analysis. The diffusive attention mechanism allows spatial structure (via the graph) and feature attention (via the Transformer) to inform each other. Compared to traditional CNN approaches, DGTN explicitly models relational dependencies by diffusing information along the image graph while preserving global contextual interactions. This architecture yields competitive classification and segmentation results on the BUSI dataset.

### 5.1. Computational Efficiency and Model Lightness

A key strength of DGTN is its computational efficiency. DGTN is designed as a lightweight, unified model without ensembling or multi-stage pipelines, enabling fast inference and a reduced memory footprint. On an NVIDIA RTX-class GPU, DGTN requires approximately 18–22 ms per image, making it suitable for real-time clinical workflows. This unified design provides a favorable trade-off between accuracy and efficiency, particularly compared to heavier cascaded pipelines.

### 5.2. Performance Gap with State-of-the-Art

Despite these advantages, DGTN’s performance remains below that of some state-of-the-art pipelines. For example, Bruno et al. [2] reported Dice ≈ 0.826 for segmentation, and Chowdary et al. [3] reported classification accuracy of 97.5%. Other recent high-performing pipelines reported in the literature also report accuracies in the 90–99% range. These approaches typically rely on multi-stage architectures, large ensembles, and extensive post-processing, which significantly increase computational cost and reduce interpretability.

In contrast, DGTN offers a single end-to-end model that jointly performs classification and segmentation, trading some absolute accuracy for efficiency, interpretability, and simplicity. The multi-class nature of BUSI (normal/benign/malignant) further increases task difficulty, particularly in distinguishing normal from benign cases. We emphasize that the parameter-count and inference-time comparisons in Table 5 are drawn from values reported in the cited literature rather than from re-implementations under identical hardware and training protocols; a controlled, head-to-head comparison against other lightweight multi-task baselines under matched conditions remains an important direction for future work.

### 5.3. Novelty and Ultrasound-Specific Adaptation

Although the diffusive attention concept was originally proposed in [8] for protein mutation prediction, this work represents, to the best of our knowledge, one of the first adaptations of bidirectional graph-Transformer diffusion to 2D ultrasound imaging and joint segmentation-classification tasks. Ultrasound-specific tuning was required, including patch-based graph construction, noise-robust preprocessing, and the gating strategy used to mitigate speckle noise and boundary ambiguity. These adaptations demonstrate that diffusion-based graph-Transformer fusion is feasible and beneficial in medical ultrasound settings.

### 5.4. Ablation and Mechanistic Insights

The ablation results indicate that both diffusion and gating are important components: removing either degrades both segmentation and classification performance. The diffusion process acts as learnable message passing, enabling spatial context propagation across weakly defined tumor boundaries, a common challenge in ultrasound imaging. The gating mechanism helps prevent over-smoothing by adaptively controlling diffusion strength across layers and samples, consistent with findings in [8].

### 5.5. Reproducibility and Robustness

Reproducibility and robustness were prioritized throughout this study. We used the open-access BUSI dataset [1] with standardized preprocessing, class balancing, and 5-fold cross-validation, and we report the fold-aggregation protocol explicitly in Section 3.3. Hyperparameters are reported in full, and all code, trained models, and data splits—including the per-fold test-set predictions underlying Table 2 and Table 3—will be released publicly to support independent verification of the statistical claims made in Section 4.5 and Section 4.6. Future work will evaluate cross-dataset generalization (e.g., Breast-Lesions-USG) and explore self-supervised pretraining to improve robustness.

### 5.6. Limitations and Future Directions

DGTN is limited by the moderate dataset size and the inherent difficulty of multi-class ultrasound classification. The statistical comparisons in Section 4.5 are similarly limited by the small number of cross-validation folds and should be extended with additional independent test data in future work. Future work will also explore larger datasets, adaptive graph learning, and extension to additional clinical tasks such as lesion detection and BI-RADS grading. Incorporating large-scale medical pretraining may further enhance generalization.

## 6. Conclusions

We presented DGTN, a Graph-Transformer hybrid architecture for joint breast ultrasound segmentation and classification. By integrating graph convolutions with Transformer attention through bidirectional diffusion and adaptive gating, DGTN captures both spatial priors and global contextual information. On the BUSI dataset, DGTN achieves 68.8% classification accuracy and a Dice score of 0.6227, demonstrating solid multi-task performance within an end-to-end framework. A key contribution of this work is a unified, lightweight, and interpretable design that achieves fast inference (18–22 ms per image) with substantially fewer parameters than typical multi-stage pipelines. While specialized ensemble-based systems achieve higher absolute accuracy, DGTN provides a favorable trade-off between performance, computational efficiency, and interpretability.

Ablation studies indicate the practical importance of diffusive attention and gating, as well as attention visualizations, provide clinically meaningful explanations of the model’s predictions. As discussed in Section 3.3 and Section 4.5, the accompanying statistical tests are based on a five-fold cross-validation design and should be read as supportive rather than conclusive evidence; we release fold-level results and code to allow independent scrutiny of these claims. All code and data splits will be made publicly available to support reproducibility. Future work will focus on scaling to larger datasets, extending to additional diagnostic tasks, and integrating self-supervised or multimodal learning to further improve accuracy and clinical applicability.

## Figures and Tables

**Table 1:** BUSI dataset distribution [1].

Class	# Images
Benign	487
Malignant	210
Normal	133
Total	780

**Table 4:** Ablation study of DGTN model variants on the primary three-class classification task, showing the impact of removing the diffusion and gating components on accuracy (%) and Dice score.

Model Variant	Accuracy (%)	Dice Score
Full DGTN	68.8	0.623
Without Diffusion	65.2	0.589
Without Gating	66.1	0.601
Without Diffusion + Gating	63.8	0.572

**Table 5:** Comparison of DGTN with baseline and multi-stage models in terms of parameter count and inference time.

Model	#Parameters (M)	Inference Time (ms)
ResNet50 + U-Net	~60	~35–45
Two-stage CNN pipeline (the typical literature)	>120	>60
DGTN (ours)	8.46	18–22

## Data Availability

This study uses the publicly available BUSI breast ultrasound dataset [1], accessible at https://doi.org/10.1016/j.dib.2019.104863. No other datasets were used.
